# Architecture and cellular composition of focal cortical dysplasia type II: qualitative review of histological studies

**DOI:** 10.3389/fncel.2025.1708220

**Published:** 2025-12-19

**Authors:** Reyes Castaño-Martín, Alice Metais, Sorana Ciura, Thomas Blauwblomme

**Affiliations:** 1 Translational Research in Neuroscience Lab, Institut Imagine, Université Paris Cité, Paris, France; 2 Institute of Psychiatry and Neuroscience of Paris, Université Paris Cité, Paris, France; 3 Service de Neuropathologie, GHU-Paris Psychiatrie et Neurosciences, Hôpital Sainte Anne, Paris, France; 4 Université de Paris Cité, Paris, France; 5 Department of Pediatric Neurosurgery Hôpital Necker, Assistance Publique Hôpitaux de Paris, Paris, France

**Keywords:** focal cortical dysplasia, dislamination, balloon cells, dysmorphic neurons, interneurons

## Abstract

Focal cortical dysplasia (FCD) is a malformation of cortical development strongly associated with drug-resistant epilepsy, particularly in children but also observed in adults. FCD type II is specifically characterised by cortical disorganisation and the presence of abnormal cells. This condition has been widely linked to hyperactivation of the mTOR signalling pathway, secondary to somatic mutations. After five decades of research, the comprehensive understanding of FCD architecture remains incomplete, with significant variability across studies, influenced by differences in tissue samples, cohort characteristics, and experimental protocols. This review aims to synthesise current knowledge on FCD architecture to clarify how the cerebral cortex is altered in FCD. We particularly focus on the hallmarks of FCD: cortical dislamination, balloon cells, and dysmorphic neurons. Additionally, we explore recent insights into the composition of cortical neuronal populations, emphasising the role of inhibitory interneuron populations, which have gained attention following discoveries regarding the involvement of GABAergic signalling in epileptogenesis. Overall, our review highlights key considerations for future single-cell and spatial studies aimed at minimising sampling bias.

## 1 Introduction

Focal cortical dysplasia (FCD) is a malformation of cortical development (MCD) described 50 years ago. Taylor et al. described in a series of lobar resections from patients with focal epilepsy a characteristic feature, the tissue harbouring “large bizarre neurons, grotesque cells probably of glial origin and a global appearance reminiscent yet distinct to tuberous sclerosis” (Taylor et al., 1971). The development of epilepsy surgery enabled to collect larger sample collections and to propose further classifications based on possible embryogenic disorders or in relation to their main histological characteristics. Barkovich and Palmini initially classified cortical malformations based on abnormalities in neuronal or glial proliferation, migration, and cortical organisation, as well as whether they were focal or generalised. This was further simplified to allow clinicians to compare more easily pharmacological and surgical outcomes (Palmini et al., 2004; Barkovich et al., 1996). In 2011, the International League against Epilepsy (ILAE) Task Force proposed a consensus classification that expanded on previous definitions. This classification refined the criteria for FCD subtypes and integrated FCD associated with other brain lesions, providing a more comprehensive framework for diagnosis and study (Blümcke et al., 2011). Finally, in 2022, the ILAE updated the classification to integrate new insights into the molecular, genetic underpinnings of FCD, particularly with the discovery of mTOR pathway mutations and neuroimaging, though the basic structure of the 2011 classification remained largely intact (Najm et al., 2022). It categorises FCD into five main types based on histopathological features and associated abnormalities and describes new entities. FCD type I encompasses isolated architectural abnormalities of cortical layering without cytological atypia and includes three subtypes: Ia, showing abnormal radial microcolumns; Ib, with tangential lamination defects; and Ic, combining both. FCD type II is defined by cortical dislamination and abnormal cells, namely IIa, dysmorphic neurons; and IIb, dysmorphic neurons and balloon cells. FCD type III presents cortical dyslamination associated with another principal brain lesion, which may contribute to epileptogenicity, such as hippocampal sclerosis (IIIa), glial or glioneuronal tumours (IIIb), vascular malformations (IIIc), or early-acquired lesions (IIId). It also recognises mild malformations of cortical development (mMCD) as a related entity, characterised by heterotopic or increased neurons in the white matter, and mMCD with oligodendroglial hyperplasia in epilepsy (MOGHE). Finally, a category of “no definite FCD on histopathology” is included for cases in which cortical disorganisation is suspected, but the findings are insufficient to meet the diagnostic criteria (Blümcke et al., 2011).

Despite the recent ILAE classification being particularly useful for diagnosis purposes, it does not consider the mechanisms of epilepsy. Earlier histopathological and clinical reviews have already emphasised the heterogeneity of findings and the challenges of standardisation (Marsan and Baulac, 2018; Blumcke et al., 2017; Sisodiya et al., 2009). Recent approaches using single-cell transcriptomics are highly susceptible to sampling bias in the tissue, and a systematic analysis of histological studies of FCD could provide a framework for canonical quantification and spatial organisation of cell populations in these malformations. As FCD classes I and III are lacking a clinic-radio-molecular signature, we chose to focus on FCD type II through a qualitative analysis of 34 studies. Moreover, as FCD is an epilepsy-associated pathology, most studies have concentrated on neuronal subtypes, which are therefore the focus of our analysis. The current review will be structured as follows: (i) histological criteria for FCD type II diagnosis according to ILAE; (ii) analysis of the global architecture of the dysplastic cortex; (iii) description and quantification of the neuronal and abnormal populations that are specific or common in FCDII.

## 2 Methods

To synthesise histological evidence on cortical architecture and cellular composition in FCD type II, we performed a qualitative review in accordance with the ENTREQ guidelines for qualitative evidence synthesis (Supplementary Table 1). We systematically searched PubMed using different combinations of relevant keywords (“focal cortical dysplasia,” FCD, “type II,” architecture, “dysmorphic neuron,” “balloon cell,” “abnormal cells,” parvalbumin, calbindin, calretinin, somatostatin, NOS, neuropeptide Y, lamination).

References were managed in Zotero. Titles, abstracts, and full texts were screened for relevance. Studies were eligible if they (i) analysed human cortical tissue diagnosed as FCD type II (per ILAE 2011 or 2022 classification, or earlier series with clear morphological correspondence); (ii) used histology, immunohistochemistry, or *in situ* hybridisation to assess cortical architecture, neuronal subtypes, or abnormal cell types (dysmorphic neurons and balloon cells); and (iii) reported qualitative or quantitative findings relevant to the review question. We included studies from 2000 to 2022, and only peer-reviewed human studies written in English were included. We excluded non-human studies, reviews or editorials, studies pooling FCD types without type II-specific data, and those lacking extractable outcomes. Furthermore, study quality and risk of bias were appraised qualitatively considering sample size, diagnosis following ILAE criteria, and methodological transparency.

The initial search yielded 358 records. Articles were screened by title/abstract, and 45 full texts were assessed for eligibility. In total, 34 studies met the inclusion criteria and were included in the qualitative synthesis. Reasons for exclusion at the full-text stage are provided in Supplementary Table 2. A prisma flow diagram of the selection process is shown in Supplementary Figure 1.

## 3 Histological characterisation according to ILAE guidelines

The ILAE diagnosis guidelines for FCD II describe in detail the expected histological abnormalities associated with the pathological analysis of the resected cortical tissue.

The main feature is the presence of dysmorphic neurons, which have unusually large cytoplasm and nucleus as well as misoriented projections. These cells still appear neuronal and can be identified using NeuN nuclei staining. A high accumulation of Nissl substance, abnormally distributed in the soma, can be a pathological characteristic of these cells. Intracytoplasmic accumulation of neurofilament proteins such as MAP2 can be detected by immunostaining. Dysmorphic neurons can be accompanied by balloon cells corresponding to large cells, possibly multinucleated, with a glassy eosinophilic cytoplasm that defines FCD type II b subgroup. These abnormal cell types can be scattered throughout the affected cortical area, including layer I, although they are more often located at the grey/white matter junction. As expected by the involvement of mTOR hyperactivity in the development of FCDII, dysmorphic neurons and balloon cells typically show intracytoplasmic pS6 accumulation as detected by anti-pS6 immunohistological staining. Cortical abnormalities associated with FCDII are characterised by a general disorganisation leading to loss of layer discrimination throughout the grey matter, with the exception of layer I. Recently described bottom of sulcus dysplasia (BOSD), which encompasses mainly FCD type IIa and IIb lesions restricted to the bottom of a sulcus, suggests the existence of different phenotypes amongst FCD type II (Najm et al., 2022; Macdonald-Laurs et al., 2024).

While the introduction of the ILAE consensus classification provided essential guidelines for the histopathological diagnosis of FCD, facilitating greater consistency among laboratories and improving comparability between studies, many of the histological investigations were conducted before 2011, with methodological differences. Furthermore, many earlier studies and some recent ones employed heterogeneous antibody panels that are not part of ILAE recommendations, reflecting specific research aims. As a result, variability between studies often arises not only from biological heterogeneity but also from methodological diversity in marker selection and interpretation criteria. Therefore, interpreting results across decades requires consideration of the evolving classification framework.

## 4 Architecture of FCD type II: dislamination and disruption of migration

The human neocortex consists of six layers (I–VI), classified according to cellular composition based on criteria such as soma size and neuronal packing density (Guy and Staiger, 2017). These layers are defined by their morphological cell types, patterns of connectivity, developmental origins, and gene expression profiles (Miyashita, 2022). They are established during development through a highly orchestrated process involving neurogenesis, neuronal migration, and layer specification through an inside-out pattern, where deeper layers form first (Rakic, 1974). Cortical neurons derive from two primary origins. Pyramidal neurons (excitatory or projection neurons) originate from radial glial cells, migrating radially from the ventricular zone (VZ) or from the subventricular zone (SVZ) to the cortical plate (Villalba et al., 2021). Radial migration of excitatory neurons is tightly regulated by signalling pathways such as Reelin and Notch (Hashimoto-Torii et al., 2008). In contrast, inhibitory interneurons arise from radial glial cells in the medial and caudal ganglionic eminences (MGE and CGE, respectively), migrating tangentially to integrate into the neocortex (Kepecs and Fishell, 2014). This process is also regulated by multiple signalling pathways, including the Eph/ephrin system and GABA/Glutamate signalling (Luhmann et al., 2015; Steinecke et al., 2014). Disruptions in these processes, whether due to genetic mutations, altered signalling pathways, or environmental disturbances, can contribute to the formation of MCDs.

One of the first identified features of FCD type II is the disruption of normal neocortical architecture (Taylor et al., 1971). Consequently, numerous studies have investigated the patterns of dislamination, cellular migration, and localisation of neuronal subpopulations in FCD type II (Fauser et al., 2013; Hadjivassiliou et al., 2010; Kuchukhidze et al., 2015; Lamparello et al., 2007; Nakagawa et al., 2017; Rossini et al., 2014; Sakakibara et al., 2012a; Table 1). Dislamination and disruption of migration are often studied together, as the loss of the six-layered structure of the cortex can arise when migrating neurons fail to reach their correct laminar positions.

**Table 1:** Dislamination and migration in FCD II.

Paper	Age	FCD II Cohort	Controls, Age	Region	Tech	Markers used	Dislamination	Loss of Neurons
Spreafico et al. (2000)	20	2			IHC	GAD, PV, GAT1	Yes, undetectable layers, only I No grey/white matter borders	Yes
Calcagnotto et al. (2005)	30,1	7	10 epileptic, 29.6 y.o.	F, T, P	IHC	CB, PV, GAD65/67	Yes	No
Alonso-Nanclares et al. (2005)	17.2	3	3 epileptic, 29.3 y.o.	P, multiple	IHC, IF	PV, NeuN	2 differentiated regions: normal-looking vs. clearly deformed, only layer I	
Lamparello et al. (2007)	4,7	10	6 epileptic, 5.2 y.o.	F, P, T, multiple	IHC	BLBP, CRMP-4, Dlx1/2, GFAP, MASH1, Otx1, Pax6, VGLUT1, VGLUT2, VGAT, P-vim, Vim	Abnormal cortical lamination and blurring of the grey–white matter junction	
			6 post-mortem, 5.8 y.o.					
Hadjivassiliou et al. (2010)	Range age: 1–52 years	9	8 Post-mortem, Range = 1 day - 17 years	F, T	IHC, IF	NeuN, Tbr1, Tbr2, Pax6, Map1b, N200, Otx1, ER81, Cux1, MASH, GFAP, CD68, CD34, CR		
			2 non-epileptic, 37 y.o.					
			4 epileptic, Range = 6–49 years					
Hanai et al. (2010)	7,6	8	5 post-mortem, 5.0 y.o.	P, F, multiple	IHC, IF	MAP2, TUJ1, Nestin, GAD, CR, CB, GFAP, Prox1, Mash1		
			2 brain tissues of 23 and 29 weeks of gestation.					
Sakakibara et al. (2012a)	2,75	9		F, P, multiple	IHC, IF	Satb2, Cutl1, Ctip2, Tbr1, Foxp1, Foxp2, Map2, Map2b, Nestin, Prox1, Gfap, Iba1, Cd68	Yes	
Fauser et al. (2013)	19,8	7	14 epileptic, 44 y.o.	F, T	IHC, ISH	CB, PV, SMI32, NeuN, TLE4, ROR*β*, ER81	No, there is basic preservation in cortical lamination	No
			3 post-mortem, 68 y.o.					
Rossini et al. (2014)	19,9	20		T, F, O, P, multiple	IHC, ISH	NeuN, SMI311, MAP2, GFAP, Vim, pS6, Cux2, Rorβ, Er81, Nurr1, CTGF	Yes, but there is partial laminar organisation of the normal looking neurons	Yes
Nakagawa et al. (2017)	19.0	22	4 post-mortem, 45.0 y.o.	F, T, O, multiple	IHC	NeuN, CB, PV, CR, SMI32, TLE4, Vim, Reelin	No	No
			7 epileptic, 34.9 y.o.					

Blank cells indicate information not reported in the study.

Regarding cortical lamination, the findings show interstudy variability, and the results are mostly qualitative. Some authors report a complete loss of lamination in FCD type II, with only the first layer discernible (Lamparello et al., 2007; Sakakibara et al., 2012a; Calcagnotto et al., 2005; Spreafico et al., 2000), as described by the ILAE criteria (Blümcke et al., 2011), while others assert that the fundamental laminar organisation is preserved, with identifiable cortical layers (Fauser et al., 2013; Nakagawa et al., 2017). Possible intra and interpatient variability of the cytoarchitectural alterations has been described (Alonso-Nanclares et al., 2005), while this is not correlated with the severity of the electrophysiological phenotype.

One aspect connected to dislamination in FCD type II is neuronal density, which could be linked to both progenitor proliferation and migration. Considerable variability is reported across various studies. Some studies report a reduction in neuron density in FCD type II lesions, such as the global reduction of the interneuron marker Glutamic acid decarboxylase (Gad) (Spreafico et al., 2000) or the reduction of *in situ* hybridisation (ISH) neuron-specific signal in different cortical layers, particularly in layer IV (Rossini et al., 2014). In contrast, other authors indicate that total neuronal population counts remain unaffected (Fauser et al., 2013; Nakagawa et al., 2017; Calcagnotto et al., 2005). However, certain studies propose that observed differences are not due to changes in the total number of neurons, but instead reflect an abnormal distribution or mislocalisation of neurons within the dysplastic cortex (Nakagawa et al., 2017; Rossini et al., 2014).

These findings suggest that the dislamination observed in FCD type II may arise from multiple factors, including an overall decrease in neuronal density, or a disruption in neuronal localisation rather than total numbers.

Another hypothesis explores dislamination as the result of a general disruption in neuronal migration. Cortical layer-specific markers (CLMs) are critical for assessing developmental and migratory disturbances, as these markers are lineage-specific proteins that reveal cellular origins. Many studies use CLMs associated with transcription factors active at various developmental stages, thus defining distinct neuronal lineages. For instance, Mash1 and Pax6 are among the earliest neurogenic regulators, playing a role in the specification of both projection neurons and interneurons in the ventricular zone (VZ). Later in development, Tbr1/2 and Otx1 mark progenitors of excitatory neurons, while Dlx1 and Dlx2 are indicative of inhibitory interneurons (Villalba et al., 2021). Other CLMs commonly used in these studies are linked to the transcriptional identity of specific cortical layers, such as RORB for layer IV, ER81 for layer V, and TLE4 for layer VI (Fauser et al., 2013; Hadjivassiliou et al., 2010; Nakagawa et al., 2017; Rossini et al., 2014). Overall, studies of CLMs in FCD II suggest an underlying issue in neuronal migration (Fauser et al., 2013; Hadjivassiliou et al., 2010; Lamparello et al., 2007; Nakagawa et al., 2017; Rossini et al., 2014; Sakakibara et al., 2012a; Hanai et al., 2010). Two studies stand out for their in-depth analysis of layer specification (Fauser et al., 2013; Rossini et al., 2014). Although both agree that normal-appearing neurons retain their laminar positions, one study reports a severely disrupted laminar structure due to the abnormal cells in FCD II. These cells are either dispersed randomly across all cortical layers or form clusters, altering the normal neuronal arrangement (Rossini et al., 2014). The other study notes differences between frontal and temporal regions, observing RORB dispersed into layer III or diminished in density, alongside reductions in ER81 and TLE4 (Fauser et al., 2013).

Thus, a key question remains: do migration defects in FCD II affect all neurons, or specifically the abnormal cells, DN, and BCs? Some studies suggest that the absence of layer-specific gene (LSG) differences implies that laminar disruption does not arise from a global migration defect, but specifically from dysmorphic neurons (DNs) and their abnormal distribution throughout the layers (Rossini et al., 2014). Conversely, other studies propose that even normal-appearing neurons in FCD may display signs of immaturity or aberrant migration, potentially contributing to synaptic dysfunction and excitability (Hanai et al., 2010; Bentivoglio et al., 2003). Furthermore, neurons in the upper cortical layers have been proposed to exhibit a more immature phenotype compared to those in deeper layers (Sakakibara et al., 2012a), suggesting a late-arrested maturation process that disrupts the strict developmental sequence of neurogenesis (Villalba et al., 2021). A potential explanation for these findings is the co-expression of glial fibrillary acidic protein (GFAP) with neuronal markers, which has been reported across studies as evidence of disruptions in the differentiation from radial glia to neurons (Hadjivassiliou et al., 2010; Lamparello et al., 2007; Sakakibara et al., 2012a; Hanai et al., 2010). While there is ongoing debate about whether this phenomenon is predominantly associated with balloon cells or dysmorphic neurons, such differentiation abnormalities may play a critical role in the disrupted cortical organisation seen in FCD II. This issue will be explored further in this study. Notably, most studies addressing cortical dislamination did not differentiate between FCD IIa and IIb, despite their distinct cellular composition. This distinction may be crucial, as recent single-cell and molecular analyses suggest subtype-specific alterations in migration and differentiation pathways (Baldassari et al., 2025; Bizzotto et al., 2025).

Altogether, FCD displays an abnormal cytoarchitecture, with loss of both columnar and laminar global patterning, which has not been quantified across patients and studies. Yet no distinctive, stereotypical pattern has been described that could explain how the migration defect occurs, and whether this is limited to abnormal mTOR-mutated cells or initiated by specific cellular populations.

## 5 Cellular composition of FCD IIb

### 5.1 FCD abnormal cells

Dysmorphic neurons (DNs) and balloon cells (BCs) are the hallmarks of FCD type II, while both cells are remarkably large, possibly as a consequence of mTOR pathway mutations known for their role in cellular growth regulation (Rossini et al., 2014; Baldassari et al., 2019; Finardi et al., 2013; Kapar et al., 2022; Lim et al., 2015; Liu et al., 2014). As a positive correlation has been established between the density of these two cell types and the somatic mutation load, their high significance in FCD pathology is currently under investigation using multiomics (Baldassari et al., 2025; Bizzotto et al., 2025; Baldassari et al., 2019).

#### 5.1.1 Dysmorphic neurons

DNs are enlarged neurons characterised by both typical neuronal markers (e.g., Neuronal Nuclei (NeuN)) and distinctive morphological features, such as a large soma (ranging from 16 to 43 microns in diameter (Blümcke et al., 2011)) and abnormal dendritic structure (Lamparello et al., 2007; Finardi et al., 2013; Szekeres-Paraczky et al., 2022; André et al., 2007). These DNs are also notable for their high neurofilament content, with markers like SMI-311, SMI-32, and N200 commonly used for their detection (Rossini et al., 2014; Baldassari et al., 2019). Histological analyses have highlighted variations in the maturity of DNs, revealing both mature neuronal markers (e.g., NeuN, MAP2, and SMI311) and undifferentiated cell markers (e.g., MAP1, Tbr1, and class III beta tubulin) (Hadjivassiliou et al., 2010; Sousa et al., 2018). Immaturity markers such as nestin and vimentin are also present in some DNs, pointing to disrupted differentiation pathways (Lamparello et al., 2007; Sousa et al., 2018). Notably, while some studies do not detect these markers in DNs, they find them in BCs (Rossini et al., 2014; Baldassari et al., 2019; Finardi et al., 2013; Table 2).

**Table 2:** Dysmorphic neurons in FCD II.

Paper	Quantif.	Soma size	Shape	pS6	NeuN	Vimentin/immature	Neuronal markers	Lineage markers	GFAP or glia	Cortical layering
Lamparello et al. (2007)	386 ± 42 DNs per ROI	113.7 μm				45% VIM 24% p-vim	82% VGLUT2 3% VGAT	76% Otx1+, 12% Pax6+, 19% BLBP+, 13% CRMP4+, 8% Mash1+, 2% Dlx2+, 7% Dlx1+	0%	
André et al. (2007)	Found in 6/10 cases FCDII; 67% of the PV/CB + neurons were cytomegalic	Cytomegalic pyramidal: 507 ± 28 μm2 Cytomegalic interneurons: 551 ± 29 μm2	Lacked an apical dendrite (not pyramidal)				GAD PV CB CR			
Hadjivassiliou et al. (2010)							Tbr1 + variable	Otx1: Cytoplasmic, perinuclear, and occasionally intranuclear. Frequently with Map1b and N200. Some labelled for Mash1. Weakly labelled for ER81. Near absence of labelled Cux1	None mentioned	Present through all cortical layers except layer I
Finardi et al. (2013)		>700 μm2	Sometimes, altered cell polarity Nissl aggregates surrounding the nuclei Thick, irregularly branched apical or basal dendrites	0		0	High SMI311		0	
Rossini et al. (2014)			Cytomegaly, abnormal dendritic arbors, and the loss of radial orientation	+	+	0	anti-SMI, anti-MAP2	Highly variable layer-specific gene expression. In the four less severely affected specimens, the DNs were prevalently localised in layers III or V/VI: Cux2, Rorβ, Er81, and Nurr1 mRNAs	0	Throughout the cortical laminae, except for layer I. Found sometimes in clusters, sometimes even in the WM
Sousa et al. (2018)			Large cell bodies and nuclei diameters Nissl substance clumped Eccentric nuclei due to filament accumulation.		+	Nestin and vimentin, and class III β-tubulin	anti-SMI-311, anti-MAP2, anti-tubulin		0	
Baldassari et al. (2019)				+		0	SMI-311			
Liang et al. (2020)							PV, Sst, and Vip			
Szekeres-Paraczky et al. (2022)		>30 microns	Cells with abnormal size and morphology				PV cytomegalic In 2 of 6 cases, DNs are surrounded by PV terminals			0
Kapar et al. (2022)			Abnormal cytoplasm and cytoplasmic extensions. Accumulation of NF isoforms is aggregated and concentrated toward the cell membrane.	ps6 + except in 2 patients	+		NF-H (phosphorylated 2F11 and non-phosphorylated SMI32)			

Blank cells indicate information not reported in the study.

Studies investigating the origins of DNs support a radial migration pattern typical of excitatory glutamatergic neurons, with high expression of Otx1 and lower levels of Mash1 or Pax6 (Hadjivassiliou et al., 2010; Lamparello et al., 2007). Although DNs are generally assigned an excitatory glutamatergic neuronal identity, evidence of inhibitory cytomegalic interneurons has surfaced (André et al., 2007; Abdijadid et al., 2014; Cepeda et al., 2014; Sakakibara et al., 2012b), underscoring the role of inhibitory systems, particularly GABA, in cortical excitability.

Electrophysiological recordings distinguish DNs by several key characteristics: higher capacitance, reduced input resistance, increased peak Calcium currents, and alterations in NMDA receptor composition, resulting in reduced sensitivity to Magnesium (Calcagnotto et al., 2005; André et al., 2008; Talos et al., 2012). Structural abnormalities in DNs, such as irregular dendritic arborisation, may foster maladaptive cortical connectivity, potentially disrupting the excitation–inhibition balance (Cepeda et al., 2003). Studies have also observed reduced glutamatergic activity alongside increased GABAergic synaptic input in DNs, with GABA puncta localised around the dysmorphic neuronal soma, highlighting the unique electrophysiological environment surrounding these cells (Shao et al., 2022). Additionally, a rhythmic pacemaker-like GABA activity, indicative of altered inhibitory interneuron behaviour, has been documented in FCD cases (Cepeda et al., 2014). In line with this hypothesis, a GABAergic-dependent depolarisation driven by chloride dysregulation supports the involvement of GABAergic mechanisms in the pathological excitation of the neuronal network (Blauwblomme et al., 2019; Bakouh et al., 2024).

In summary, DNs are implicated in the abnormal excitability characteristic of FCD, with their unique morphology and electrophysiological features potentially influencing seizure generation and propagation (Lee et al., 2022; Rampp et al., 2021).

#### 5.1.2 Balloon cells

BCs exhibit specific traits, particularly in their glial-like immature phenotype and distinctive localisation. These unusually large, rounded cells are specific to FCD IIb and exhibit similarities to the giant cells found in Tuberous Sclerosis Complex (TSC) (Najm et al., 2022).

Quantifying BCs remains challenging due to variability among studies, with estimates ranging from 0.05 to 68 BCs per mm^2^ and soma diameters between 39 to 127 microns (Lamparello et al., 2007; Baldassari et al., 2019; Finardi et al., 2013). Most studies locate BCs in the white matter or at the white matter–grey matter junction, in deep cortical layers (Rossini et al., 2014; Spreafico et al., 2000; Finardi et al., 2013; Szekeres-Paraczky et al., 2022; Thom et al., 2003). However, a few studies describe BCs as present across all cortical layers (Alonso-Nanclares et al., 2005; Sousa et al., 2018; Table 3).

**Table 3:** Balloon cells in FCD II.

Paper	Quantif.	Soma size	Morphology	pS6	NeuN	Vimentin/immature	Neuronal markers	Lineage markers	GFAP	Region	Electric activity
Spreafico et al. (2000)			Binucleated elements with ill-defined somatic profiles							WM	
Aronica et al. (2003)			Eccentric nuclei and ballooned opalescent eosinophilic cytoplasm.				MAP2. Diff. expression of the 3 mGluR		Some GFAP+		
Thom et al. (2003)			Binucleate				Some CB + seemed BCs			WM	
Alonso-Nanclares et al. (2005)			Large size, and rounded shape, ill-defined cell membrane, one or more eccentric nucleoli. Nissle, accumulation of filaments							Scattered throughout the neocortex	No synapses identified
Lamparello et al. (2007)	291 ± 37 cells/cm2	12661.26 microns2	Ovoid shape, enlarged cell soma, and laterally displaced nucleus			83% BCs vimentin and 66% p-vim	VGLUT2 + but VGAT-	Otx1 BLBP and Pax6 MASH1 in a small number	GFAP 50%		
Hadjivassiliou et al. (2010)			Some multinucleated		NeuN-		Tbr2	Strong nuclear labelling for Pax6 in 4/9 cases with co-localisation with GFAP but not NeuN. The majority of cases were labelled Otx1, and there was some co-expression with GFAP and CD34. Intense nuclear positivity for ER81. Majority labelled Cux1	GFAP+ CD34+		
Finardi et al. (2013)	0.8 to 6.9 cells/ 0,1 mm2		Large, malformed cells of uncertain origin that were frequently multinucleated	+		Vim+			Mainly GFAP-	Grey/WM junction.	
Rossini et al. (2014)			Voluminous cytoplasms, displaced nuclei, prevalently glial-immature	+		Vim+, Nestin+			ND	Always found in the white matter	
Sousa et al. (2018)			Oval, large cell body, a large, pleomorphic, and eccentric nucleus, and a prominent nucleolus. No Nissl substance. Often several nuclei		NeuN+	Vim+, Nestin+, neuronal class III β-tubulin+	MAP2+, SMI311+, Beta-tubulin+		GFAP+	All cortical depths, mostly WM and the grey/WM transition.	
Baldassari et al. (2019)	0.05 to 8.42 cells/mm2	Longest cell body mean = 39 μm	Large, ovoid-shaped cell body, often presenting an eccentric nucleus or multiple nuclei	+		Vim+	SMI311-?				
Liang et al. (2020)							PV, Sst, Vip				
Szekeres-Paraczky et al. (2022)			Rounded bodies, few thin processes						GFAP+	WM	
Kapar et al. (2022)			Large, homogenous, opaque, eosinophilic cytoplasm and an eccentrically located nucleus. The Nissl body cannot be seen	Weak, 2neg	NeuN-		NF-H-				

Blank cells indicate information not reported in the study.

BCs exhibit distinct morphological features, including a binucleated or multinucleated profile, a rounded or ovoid shape, sparse thin processes, and an accumulation of cytoplasmic filaments (Hadjivassiliou et al., 2010; Lamparello et al., 2007; Rossini et al., 2014; Spreafico et al., 2000; Alonso-Nanclares et al., 2005; Baldassari et al., 2019; Finardi et al., 2013; Kapar et al., 2022; Szekeres-Paraczky et al., 2022; Sousa et al., 2018; Thom et al., 2003; Aronica et al., 2003). These filaments are identified as vimentin, and together with the presence of nestin, indicate BCs’ immature phenotype (Rossini et al., 2014; Sousa et al., 2018). Although BCs are primarily characterised by a glial-like phenotype and express GFAP, two studies reported BCs expressing neuronal markers, such as NeuN (Sousa et al., 2018) and MAP2 (Sousa et al., 2018; Aronica et al., 2003), suggesting a possible dual nature or disrupted maturation. Additionally, markers of interneurons such as calbindin, parvalbumin, somatostatin, and NPY have been detected in BCs (Thom et al., 2003; Liang et al., 2020).

Similar to DNs, BCs display signs of abnormal migration and maturation. Pax6 and Otx1 are present in BCs, alongside smaller amounts of Mash1 and BLBP, indicating a radial glial origin and suggesting a developmental arrest. A comparative study using an extended protein marker panel revealed distinctions between BCs and DNs, where DNs often express N200, Map1b, and Tbr1, while BCs are marked with Tbr2 and Cux1. These findings suggest they may derive from different migratory progenitor subpopulations (Hadjivassiliou et al., 2010).

Electrophysiologically, BCs are largely inert, with no evidence of synaptic connections and lacking both voltage-gated and Na + currents (Alonso-Nanclares et al., 2005; Cepeda et al., 2006).

Despite their inactivity, their astroglial characteristics may contribute to the epileptogenic environment in FCD, as proposed by some studies (Cepeda et al., 2006).

### 5.2 Neuronal populations

Neurons in the brain are broadly classified as excitatory (primarily pyramidal neurons) or inhibitory (interneurons). Given the known imbalance of excitation and inhibition in FCD, multiple studies have focused on these neuronal populations, with particular interest in interneuron subtypes and their roles in FCD-related epileptogenicity.

#### 5.2.1 Excitatory neurons

In excitatory neurotransmission, two primary glutamate receptor families have been identified: the metabotropic glutamate receptors (mGluRs) and the N-methyl-D-aspartate (NMDA) ionotropic receptors. The mGluRs, which are G-protein-coupled receptors, play a modulatory role in synaptic transmission (Niswender and Conn, 2010), while NMDA receptors are critical for fast excitatory signalling (Hansen et al., 2021). Enhanced excitatory transmission has been consistently observed in FCD, with studies highlighting alterations in the expression, functionality, and subunit composition of Glu receptors. Specifically, group I mGluRs (mGluR1α and mGluR5) show strong expression in DNs and BCs, with additional expression of mGluR2/3 also noted in BCs (Aronica et al., 2003). Additionally, structural variations in NMDA receptors, such as a decrease in the NR2B subunit, have been reported in FCD neurons, which correlate with altered electrophysiological properties (André et al., 2004).

#### 5.2.2 Inhibitory neurons

*γ*-Aminobutyric acid (GABA), the main inhibitory neurotransmitter in the cortex, is essential for maintaining excitation–inhibition balance. Synthesised in the axons of GABAergic interneurons, GABA is released to modulate cortical excitability and synchrony (Treiman, 2001). Cortical interneurons account for approximately 25% of total cortical neurons (Wonders and Anderson, 2006) and, beyond their role in neurotransmission, are also implicated in key developmental processes such as cell proliferation and migration (Owens and Kriegstein, 2002). Some studies indicate an overall reduction in GABAergic neurons in FCD, evidenced by a decrease in glutamic acid decarboxylase (GAD), a key enzyme and marker for GABA synthesis (Calcagnotto et al., 2005; Spreafico et al., 2000; Sakakibara et al., 2012b).

GABAergic interneurons are further categorised based on their expression of specific proteins and neuropeptides. Major subtypes include those expressing Parvalbumin (PV), Somatostatin (SST), Vasoactive Intestinal Peptide (VIP), Nitric Oxide Synthase (NOS), Reelin, Neuropeptide Y (NPY), Calretinin (CR), and Calbindin (CB), though overlap in these markers is common (Kepecs and Fishell, 2014). The loss or impairment of these GABAergic interneurons disrupts inhibitory control over cortical pyramidal neurons, fostering hyperexcitability and contributing to epileptogenesis (Valencia et al., 2006).

##### 5.2.2.1 Parvalbumin

Parvalbumin (PV) is a calcium-binding protein widely expressed in a major subtype of GABAergic interneurons, recognised for their potent inhibitory role in cortical circuits. These neurons are primarily localised in layer IV of the human cortex, though they are also present in adjacent layers (Nakagawa et al., 2017). PV-expressing interneurons are particularly effective in modulating calcium signalling within synapses, impacting the duration and amplitude of calcium transients and thus directly regulating excitability (Kuchukhidze et al., 2015; Zamecnik et al., 2006). A total of 26 studies were assessed for PV interneuron alterations in FCD, with 14 specifically examining this subpopulation and 6 studies quantifying their density (Table 4). Three studies were excluded from further analysis as they lacked data specific to FCD type II or combined FCD types I and II without distinction (Fauser et al., 2013; Kuchukhidze et al., 2015; Finardi et al., 2013).

**Table 4:** Quantification papers on parvalbumin-positive neurons in FCD II.

Paper	Mean age	FCD II Cohort	Controls	Region	Tech	Quantification	Markers	Results	PV
Zamecnik et al. (2006)	16,8	5	7 post-mortems Mean age = 32.0	1 F 2 T 2 P	IHC	20 ROI, 4X, total number of cells, number of cells/ layers; percentage of control values	PV	PV immunoreactivity was disrupted and almost random. Density of PV-IR in FCD II is 12.2% of control values. PV cell quantification: FCDII: 5.9 ± 0.8 vs. Ctrl: 48.2 ± 1.1. Decrease of PV processes (more in FCDII, not quantified only seen)	↓
Sakakibara et al. (2012a)	5	4	7 post-mortem Mean age = 1.9	F	IHC	Number of cells counted in 5 fields and corrected per 100 nuclei in each region	CR, CB, GAD, PV, NPY	PV FCDII: 3.6 ± 2.7% vs. Control: 7.6 ± 6.5% No significant difference for PV, GAD also diminished	
Medici et al. (2016)	17,6	12	4 epileptic cryptogenic (poor surgical outcome)	6 F 2 O 3 T 1 P	IHC	Series of contiguous images was captured at 10x (4 mm2) in the grey matter, and markers were manually counted and averaged	GFAP, SMI311, NeuN, Vim, PV	Some PV is abnormally large, with no significant changes in PV. Rearrangement but no quantitative difference. FCDII: 23 cells/mm2 (19% of GAD+) vs. Ctrl: 42 cells/mm2 (25% of GAD+)	
Nakagawa et al. (2017)	19	22	4 post-mortems Mean age = 45.0	13 F 4 T 1 O 2 P-O 1 F-T 1 T–P 1 T–P-O	IHC with HRP	Total neuron densities + layer densities were determined by counting the cells in NeuN sections from the WM to the pial surface. Cell counting software: cells/10^4mm2	NeuN, CB, PV, CR, SMI32, TLE4, VIM, RELN	PV density in FCD IIb and epilepsy controls compared to autopsy. Moreover, less arborization and axonal plexus. FCDII: 0.09 cells/ 10.000 μm2 vs. Control: 0.29 cells/ 10.000 μm2. In layer IV, the change is from 0.65 to 0.2. Age: PV accentuated the decrease difference in young children	↓
			7 epileptic Mean age = 34.9						
Liang et al. (2020)	11,9	19	5 benign tumour Mean age = 10.4	7 F 2 T 2 P 4 F-P 1 F-T 1 P-O 1 F-T–P 1 F-T-O	IHC	Counting in the superficial and deep part of the cortex. 4x photos were taken, corresponding to an area of 3,481 microm x 2621microm, which was gridded to regions of 10×10, therefore areas of 348.1 microm x 262.1 microm. They were selected from the pia to the GWM junction. Cells counted manually.	PV, SST, VIP	FCDIIb presented the highest decrease in PV PV density in superficial: - Ctrl: 200 cells/mm2 - FCD IIa: 150 cells/mm2 - FCD IIb: <100 cells/mm2 PV density in deep cortex: - Ctrl: 200 cells/mm2 - FCD IIa: 180 cells/mm2 - FCD IIb: <100 cells/mm2” PV is also present in abnormal cells, DNs, and BCs	↓
			3 post-mortems Mean age = 10.4						
Szekeres-Paraczky et al. (2022)	33,75	6	5 post-mortems Mean age = 61.0	4 F 1 O 1 P	IHC frozen sections	Quantification of PV-terminals in layer III and V around NeuN. Quantification only in regions of distinguishable layers. PV-NeuN double staining was excluded from the quantification.	NeuN, SMI32, PV, GFAP	Significantly increased PV + terminals in layers III and V: Layer III: FCD 3,43/cell 8(1,122 terminals/cell) vs. Ctrl 1.76/cell (591 terminals in 336 cells) Layer V: FCD 2,12/cell (702 terminals/331 cells) vs. Ctrl 0,96/cell (320 terminals/332 cells) 4 of 6 cases: mostly, normally sized cells were found, but they showed more numerous PV-immunopositive terminals 2 of 6 cases: DNs are surrounded by PV terminals	↑

Blank cells indicate information not reported in the study.

In total, six studies report a reduction in PV expression in FCD (Nakagawa et al., 2017; Spreafico et al., 2000; André et al., 2008; Thom et al., 2003; Liang et al., 2020; Zamecnik et al., 2006), while the other five report no significant change or describe altered distribution patterns of PV without quantitative loss (Calcagnotto et al., 2005; Alonso-Nanclares et al., 2005; Finardi et al., 2013; Sakakibara et al., 2012b; Medici et al., 2016). On the other hand, PV density in FCD is increased in two studies (Kuchukhidze et al., 2015; Szekeres-Paraczky et al., 2022). Interstudy variation included: sample size (range 2–22 patients), patient age (range: 0.19–48 years), and sample locations, despite the frontal cortex being most commonly explored. Control groups were also not standardised: post-mortem tissue (4 studies), epileptic controls (5 studies), both or non-epileptic cortex (2 studies).

In summary, the majority of the evidence suggests a potential reduction in PV interneurons in FCD; however, the heterogeneity of patient samples, regional analyses, and control methodologies complicate definitive conclusions (Table 4).

##### 5.2.2.2 Calbindin

Calbindin (CB) is another calcium-binding protein commonly found in layers II/III of the human cortex. Similar to parvalbumin-expressing interneurons, studies on CB-expressing neurons in FCD exhibit high variability (Table 5). Our review identified eight papers investigating CB expression in FCD, but only five were included despite interstudy variability in age (range: 0.19–46 years) and control samples (epileptic tissue n = 2; both post mortem and epileptic cortex n = 1); only 2 of 5 studies provided objective quantification of their findings.

**Table 5:** Histological findings on calbindin-positive neurons in FCD II.

Paper	Age	FCD II Cohort	Controls	Region	Quantif./Tech	Markers	Results	CB
Thom et al. (2003)	12,8	12	8 post-mortem Mean age = 59	9 F 2 T 1 hemispherectomy	No quantification IHC	CR CB PV NPY Reelin	7 of 12 cases of FCD showed CB + DNs Some CB + seemed BCs 10 of 12 cases showed a decrease in CB + neurons in the FCD region	↓
			Normal-looking cortex adj. to the FCD					
Calcagnotto et al. (2005)	30,1	7	10 epileptic Mean age = 29.6	4 F 12 T 1 P	No quantification IHC	CB PV GAD65/67	CB showed an abnormal distribution throughout all cortical layers (vs. their normal localisation in layers II/III in controls) Appeared to be usually scarce and in small clusters in some areas GAD neurons were reduced, but normally distributed	
André et al. (2007)	1,25	11	6 epileptic Mean age = 6.6	F and T	No quantification IHC	GAD CB PV CR	Severe CD: CB in Layer II is relatively preserved and of normal size Abnormally large cells in layers IV-VI, bipolar or multipolar 67% of the PV/CB + neurons were cytomegalic in layers V-VI	
Sakakibara et al. (2012a)	5	4	7 post-mortem Mean age = 1.9	F	IHC: Number of cells counted in 5 fields and corrected per 100 nuclei in each region	CR CB GAD PV NPY	Less GAD+: Control: 4.8 ± 0.8% vs. FCDII: 1.8 ± 0.6% Less CB+: Control: 6.0 ± 2.2% vs. FCDII: 2.8 ± 2.1% Significantly different for CB	↓
Nakagawa et al. (2017)	19	22	4 post-mortem Mean age = 45	13 F 4 T 1 O 5 multiple	IHC with HRP: Total neuron densities + layer densities were determined by counting the cells in NeuN sections from the WM to the pial surface. Cell counting software: cells/10^4mm2	NeuN Reelin CB PV CR SMI32 TLE4 Vim	Not altered layer-specific density in layer II/III. CB Significant decrease in FCD2b and epileptic control compared to post-mortem control. No reduction in NeuN-positive neurons Control: 0.25 cells/ 10.000 μm2 FCDII: 0.16 cells/ 10.000 μm2 Quantification of total calbindin was not significant in layer II. Age: no difference in CB cell density in young children	↓
			7 epileptic Mean age = 34.9					

Blank cells indicate information not reported in the study.

In total, three of the five studies (Nakagawa et al., 2017; Sakakibara et al., 2012b; Thom et al., 2003) found a reduction in CB-positive neurons in FCD II. By contrast, in 2 of 5 reports, CB neuron density was not modified despite an abnormal distribution across cortical layers (Calcagnotto et al., 2005; André et al., 2007).

Overall, although data suggest a potential reduction in CB-positive interneurons within cortical layers in FCD II, limited sample sizes and cohort heterogeneity hinder definitive conclusions.

##### 5.2.2.3 Calretinin

Calretinin (CR) is a calcium-binding protein whose cellular functions remain poorly understood, although it is believed to play a role in buffering intracellular calcium and modulating neuronal excitability (Dargan et al., 2004). In the neocortex, CR is predominantly located in layers II and III, with evidence suggesting that the morphology and function of CR-expressing neurons may vary by brain region, potentially reflecting adaptations related to epilepsy (Tóth et al., 2010). Interestingly, both decreases and increases in CR expression have been proposed as neuroprotective mechanisms against cytotoxicity (Lukas and Jones, 1994; Turner et al., 2007).

Our literature review identified seven studies investigating CR interneurons in the context of FCD, of which five were included due to their focus on FCD II (Table 6).

**Table 6:** Histological findings on calretinin-positive neurons in FCD II.

Paper	Age	FCD II cohort	Controls	Region	Quantif./Tech	Markers	Results	CR
Thom et al. (2003)	12,8	12	8 post-mortem Mean age = 59	9 F 2 T 1 hemispherectomy	No quantification IHC	CR CB PV NPY Reelin	4 of 12 cases showed a reduction in CR+ 3 of 12 cases showed increased CR + in WM	↓
			Normal-looking cortex adj. to the FCD					
André et al. (2007)	1,25	11	6 epileptic Mean age = 6.6	F and T	No quantification IHC	GAD CB PV CR	Severe CD: CR preserved in layer II, although the regions were very disorganised and with cytomegalic pyramidal neurons, showing absence of CR+ No hypertrophic cells labelled CR + .	
Barinka et al. (2010)	19,5	8	9 post-mortem Mean age = 55	3 F 4 T 1 O	IHC: using an image analysis software, 10X objective. Total neuron densities + layer densities counted as number of cells/ mm2	CR	Dysmorphic neurons and balloon cells were CR- The overall distribution and morphology were conserved CR + same localisation, but the intensity is lower CR + decreased (significant), but no difference between type IIa and IIb FCDII count: 16.7 ± 1.0; representing 48.7% control	↓
Sakakibara et al. (2012a)	5	4	7 post-mortem Mean age = 1.9	F	IHC: Number of cells counted in 5 fields and corrected per 100 nuclei in each region	CR CB GAD PV NPY	Less GAD+: Control: 4.8 ± 0.8% vs. FCDII: 1.8 ± 0.6% Less CR+: Control: 10.6 ± 4.3% vs. FCDII: 2.8 ± 2.2% Significantly different for CR	↓
Nakagawa et al. (2017)	19	22	4 post-mortem Mean age = 45	13 F 4 T 1 O 5 multiple	IHC: Total neuron densities + layer densities were determined by counting the cells in NeuN sections from the WM to the pial surface. Cell counting software: cells/10^4mm2	NeuN, Reelin, CB, PV, CR, SMI32, TLE4, Vim	No histologic abnormalities or differences in cell density. No reduction in NeuN-positive neurons. Control: 0.35 cells/ 10.000 μm2. FCDII: 0.25 cells/ 10.000 μm2. Age: no difference in CR cell density in young children	
			7 epileptic Mean age = 34.9					

Blank cells indicate information not reported in the study.

The data suggest a potential reduction of CR-expressing interneurons in FCD II. The variability across studies and the heterogeneity of the patient cohorts complicate the ability to draw definitive conclusions.

##### 5.2.2.4 NPY

The remaining interneuron subtypes, including Neuropeptide Y (NPY)-expressing neurons, are comparatively under-researched in the context of FCD. NPY is a neurotransmitter abundantly expressed in the central nervous system, where it plays a role in modulating neuronal activity and cortical excitability (Vezzani et al., 1999). Known as a potent endogenous anticonvulsant, NPY is released during high-frequency stimulation, with studies showing increased NPY fibre density in hippocampal epilepsy models (Furtinger et al., 2001).

In 2003, Thom et al. investigated NPY expression in FCD, finding an increase in NPY fibres in the superficial cortex, particularly in layers I and II, which they proposed as a mechanism to counteract cortical excitability. These data were confirmed in 3 other studies despite interstudy variability regarding patient age (range 1–36), and quantification method (NPY fibre density versus NPY neuronal density) (Sakakibara et al., 2012b; Thom et al., 2003; Li et al., 2016; Table 7).

**Table 7:** Histological findings on NPY-positive neurons in FCD II.

Paper	Age	FCD II cohort	Controls	Region	Quantif./Tech	Markers	Results	NPY
Thom et al. (2003)	12,8	12	8 post-mortem Mean age = 59	9 F 2 T 1 hemispherectomy	No quantification IHC	CR CB PV NPY Reelin	Increase in density of the NPY plexus in superficial cortex: NPY fibre length ratios (mean 2, range 1.8–6.1) compared to control lobes (mean 0.98, range 0.6–1.5) Variable results on the dysplastic area, intense labelling of the DNs 4 of 12 cases showed an increase in NPY cells. NPY fibre length was decreased in the dysplastic area versus the adjacent area	↓
			Normal-looking cortex adj. to the FCD					
Sakakibara et al. (2012a)	5	4	7 post-mortem Mean age = 1.9	F	IHC: Number of cells counted in 5 fields and corrected per 100 nuclei in each region	CR CB GAD PV NPY	Less GAD+: Control: 4.8 ± 0.8% vs. FCDII: 1.8 ± 0.6% Less NPY+: Control: 0.7 ± 0.6% vs. FCDII: 0.2 ± 0.1% Not significantly different for NPY	
Li et al. (2016)	11,4	21	5 post-mortem Mean age = 48.8	9 F 1T 11P	IHC	GAPDH NPY Y1R Y2R Y5R	Striking increase in NPY fibre plexus: parietal lobe (by 31.2%), in temporal (by 61.4%), and in frontal (by 46.3%) Also by WB and RT-PCR”	↑

Blank cells indicate information not reported in the study.

These findings collectively support an anticonvulsant role for NPY and suggest its potential as a therapeutic target for seizure management.

##### 5.2.2.5 Reelin

Reelin (RELN) is an extracellular matrix serine protease that plays a critical role in the neuronal layering of the cerebral cortex during development. While RELN is widely associated with Cajal–Retzius cells, a subset of RELN-expressing interneurons also persists in the upper cortical layers into adulthood (Vílchez-Acosta et al., 2022). Due to its involvement in neuronal migration, RELN is of particular interest in studies of FCD II, as alterations in cortical layering and cell positioning are characteristic of this condition.

Despite this, only two studies have focused on RELN-expressing neurons in FCD II, with both reporting no significant differences (Table 8) in RELN cell density compared to controls (Nakagawa et al., 2017; Thom et al., 2003).

**Table 8:** Histological findings on Reelin-positive neurons in FCD II.

Paper	Age	FCD II cohort	Controls	Region	Quantif./Tech	Markers	Results	RELN
Thom et al. (2003)	12,8	12	8 post-mortem Mean age = 59	9 F 2 T 1 hemispherectomy	IHC: only quantification in Cajal–Retzius cells 20X, reelin positive cells/mm2	CR CB PV NPY Reelin	Reelin was always in layer I in ctrl, MD, and FCD; occasionally, smaller neurons in other layers were not quantified. Reelin is higher in FCD but not significantly. 0.26 ± 0.13 cells/mm in adult controls 0.62 ± 0.66 cells/mm in FCD 0.65 ± 0.44 cells/mm in the adjacent cortex FCD	
			Normal-looking cortex adj. to the FCD					
Nakagawa et al. (2017)	19	22	4 post-mortem Mean age = 45	13 F 4 T 1 O 5 multiple	IHC with HRP: Total neuron densities + layer densities were determined by counting the cells in NeuN sections from the WM to the pial surface. Cell counting software: cells/10^4mm2	NeuN Reelin CB PV CR SMI32 TLE4 Vim	Always in layer 1 No significant difference	
			7 epileptic Mean age = 34.9					

Blank cells indicate information not reported in the study.

##### 5.2.2.6 VIP, SST, and NOS

Among the less-studied interneuron subtypes in FCD II, only a single paper each addresses vasoactive intestinal peptide (VIP), somatostatin (SST), and nitric oxide synthase (NOS) interneurons, making it difficult to draw definitive conclusions. VIP and SST interneuron quantification was not modified in FCD (Liang et al., 2020), while NOS interneurons density was increased (González-Martínez et al., 2009).

As a summary, a deregulation of GABAergic system in relation to variation of the density distinct interneuron population is proposed, despite high interstudy variability biases generalisation of this hypothesis.

## 6 Glia

Although the present review primarily focuses on neuronal populations and abnormal cell types, it is important to note that astrogliosis and microglial activation are well recognised histopathological features of FCD II (Blümcke et al., 2011; Boer et al., 2006; Galvão et al., 2023). Reactive astrocytes, identified by GFAP or vimentin immunoreactivity, are commonly distributed throughout the dysplastic cortex and white matter. A subset of the studies included in this review has also provided specific information on glial alterations (Lamparello et al., 2007; Finardi et al., 2013; Szekeres-Paraczky et al., 2022; Aronica et al., 2003). While one of the studies identified reactive astrocytes in both FCD IIa and FCD IIb (Aronica et al., 2003), other studies only focused on FCD IIb, showing a striking increase in astrogliosis (Lamparello et al., 2007; Finardi et al., 2013; Szekeres-Paraczky et al., 2022), with two of them reporting hypertrophic or dysmorphic astrocytes in FCD IIb (Lamparello et al., 2007; Finardi et al., 2013; Galvão et al., 2023). Interestingly, recent single-cell data have shown that this increase occurs only in FCD IIb, suggesting a distinct molecular phenotype compared to FCD IIa (Galvão et al., 2023), and therefore opening the question of whether the BCs might play a role in this gliosis. Finally, studies have shown that gliosis might play a key role in epileptogenesis (Patel et al., 2019), with the extent of gliosis correlating with neuronal loss and epilepsy duration (Finardi et al., 2013).

Overall, glial reactivity appears to be a consistent component of the cellular landscape in FCD type II, where glial remodelling may influence neuronal excitability and contribute to the maintenance of the epileptogenic network.

## 7 Future perspectives and guidelines

Given the pronounced heterogeneity of FCD lesions and the variability across histological reports, future studies could benefit from standardised approaches to tissue sampling. We propose that single-cell and spatial transcriptomic analyses should prioritise the use of comparable cohorts in terms of patient age and cortical region, and ensure that the dissected areas encompass the pathological core containing abnormal cells. Because FCD lesions are focal and abnormal cells may be scarce, spatially resolved methods such as high-resolution spatial transcriptomics or multiplex imaging offer valuable strategies to confirm lesion localisation and guide tissue selection. Moreover, as most studies currently rely on frozen nuclei preparations due to the fragility of epileptic tissue and large neuronal size, attention should be given to maintaining cellular integrity and representativeness across all cortical layers, from pia to white matter. Distinguishing between FCD IIa and IIb in both histological and molecular analyses will also be essential to disentangle subtype-specific developmental and epileptogenic mechanisms.

Recent advances in single-cell and spatial transcriptomic technologies are beginning to reveal the molecular complexity of FCD type II, complementing the extensive histological evidence accumulated over the past decades. Unbiased single-cell RNA sequencing and nuclei transcriptomics have identified distinct neuronal, glial, and abnormal cell populations within FCD lesions, highlighting both inter- and intra-lesional heterogeneity (Baldassari et al., 2025; Bizzotto et al., 2025). Spatial transcriptomic approaches are further enabling the contextualization of these molecular signatures within the dysplastic cortex, helping to localise abnormal cell clusters and their interactions with the surrounding microenvironment (Wang et al., 2024). Furthermore, computational strategies such as cell-type deconvolution from bulk RNA-seq data have also emerged as complementary tools for estimating cellular composition in epileptogenic tissue, with studies confirming neuronal loss and prominent astrogliosis, in line with histological observations (Galvão et al., 2023).

Altogether, integrating histological and molecular approaches, while ensuring rigorous sampling strategies and cortical layer coverage, will be essential to build a comprehensive and unbiased understanding of the cellular architecture and pathophysiology of FCD type II.

## 8 Conclusion

FCD type II represents a heterogeneous condition that challenges our understanding of cortical development and epileptogenesis. If the diagnosis is consensual with cortical disorganisation and the presence of balloon cells and dysmorphic neurons, in-depth microarchitecture and cellular quantification are still underway. Histological and electrophysiological studies acquired during the last decades are a milestone for further interpretation of multiomic data that will help take into account interpatient and spatial variability of the tissue.
