# Supplementary material for: Architecture and cellular composition of focal cortical dysplasia type II: qualitative review of histological studies
Source: Front Cell Neurosci. 2025 Dec 19;19:1708220. doi: 10.3389/fncel.2025.1708220 (PMC12757251; doi:10.3389/fncel.2025.1708220)
Supplement: Supplementary file 1 [file Table_1.docx]

Supplementary Material

# Supplementary Figures and Tables


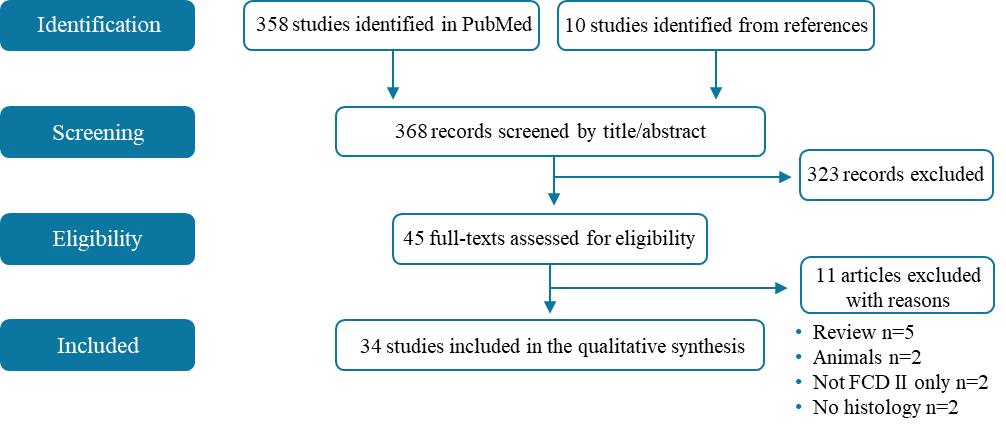


**Supplementary Figure 1.** Prisma flow diagram of the selection process

**Supplementary Table 1.** ENTREQ checklist guidelines

| **No** | **Item** | **Description** |
| --- | --- | --- |
| **1** | Aim | To synthesise histological evidence on cortical architecture and cellular composition in FCD type II. |
| **2** | \| Synthesis methodology \| \| --- \|  \|  \| \| --- \| | Qualitative review |
| **3** | Approach to searching | Pre-planned systematic PubMed search using defined keywords. |
| **4** | Inclusion criteria | Inclusion = human FCD II tissue, histology/IHC/ISH, qualitative or quantitative outcomes; Exclusion = non-human, reviews/editorials, pooled FCD without type II data, no extractable results. |
| **5** | Data sources | PubMed database; as it has the largest biomedical index for relevant histological studies. |
| **6** | Electronic search strategy | Search terms listed: “focal cortical dysplasia”, FCD, “type II”, architecture, “dysmorphic neuron”, “balloon cell”, “abnormal cells”, parvalbumin, calbindin, calretinin, somatostatin, NOS, neuropeptide Y, lamination |
| **7** | Study screening methods | 358 records identified, screened by title/abstract, 45 full-text reviewed; reasons for exclusion provided (Supplementary Table 2). |
| **8** | Study characteristics | 34 included studies were published between 2000 and 2022, with cohorts ranging from 2 to 22 FCD II patients, and controls comprising post-mortem tissue, epileptic and non-epileptic resections, and tumour samples. Patient ages ranged from infancy to middle age, most studies examined frontal and temporal lobes, and methodologies included IHC, IF, ISH, and molecular analyses, with heterogeneous quantification strategies. |
| **9** | Study selection results | PRISMA-style flow shown in Supplementary Fig. 1. |
| **10** | Rationale for appraisal | Selection limited to peer-reviewed human histological studies; robustness ensured by inclusion criteria rather than formal quality scoring. |
| **11** | Appraisal items | Not formally scored; appraisal based on study design relevance and FCD II specificity (as described in Methods). |
| **12** | Appraisal process | Not applicable |
| **13** | Appraisal results | Not applicable |
| **14** | Data extraction | Extracted qualitative/quantitative findings on architecture, abnormal cells, neuronal subtypes, interneuron markers; presented in narrative form with tables |
| **15** | Software | Zotero for reference management |
| **16** | Number of reviewers | First author with supervision of co-authors |
| **17** | Coding | No inductive line-by-line coding was performed. Instead, a deductive categorisation framework based on histological domains of interest (cortical architecture, dysmorphic neurons, balloon cells, and neuronal subtypes) was used. Findings from each study were extracted and assigned to these categories to enable comparison across studies. |
| **18** | Study comparison | Differences in methodology, sample size, and control tissue noted. |
| **19** | Derivation of themes | Themes were derived deductively, guided by established histopathological domains of FCD type II. Findings from each study were first grouped under codes reflecting specific observations (e.g., interneuron density, DN morphology, BC markers), and then synthesised into broader themes: (i) histological criteria for diagnosis, (ii) cortical architecture and delamination, (iii) abnormal cell types, and (iv) neuronal populations (with subthemes for interneuron subtypes). |
| **20** | Quotations | Not applicable |
| **21** | Synthesis output | The review found that FCD II is consistently characterised by cortical disorganisation, delamination, and the presence of dysmorphic neurons and balloon cells, alongside evidence of interneuron deregulation (notably PV, CB, CR). Despite interstudy variability, the overall synthesis points to a disrupted excitatory–inhibitory balance as a key feature of FCD II. |

**Supplementary Table 2.** Reasons for exclusion at full-text stage

| **First author, year** | **Reasons for exclusion** |
| --- | --- |
| Sisodiya, 2009 | Review |
| Jansen, 2010 | No histological results |
| Abdijadid, 2014 | Review |
| Crino 2015 | Review |
| Kuchukhidze, 2015 | Focus on FCD I and III |
| Blumcke 2017 | Clinical statistical study |
| Pfisterer 2020 | All epilepsy |
| Zhong, 2020 | Done in mice |
| Jesus-Ribeiro, 2021 | Review |
| Lee, 2022 | Review |
| Zheng, 2023 | Done in rats |
